# Equipment of Texas Medical College and Hospital

**Published:** 1888-09

**Authors:** H. A. West

**Affiliations:** Galveston, Texas


### EQUIPMENT OF TEXAS MEDICAL COLLEGE AND HOSPITAL.

### Letter from Prof. H. A. West, M. D.

GALVESTON, TEXAS, Sept. 12, 1888.

*Editor Daniel's Texas Medical Journal:*

As mention was made in the last number of DANIEL'S T. M. JOURNAL of the fact that I had been selected by the Faculty of the Texas Medical College and Hospital to proceed to Philadelphia to purchase an outfit for the college, and also to engage the services of a competent man to teach histology, pathology and bacteriology, in compliance with a promise made before my departure I will give the readers of the JOURNAL some account of the successful accomplishment of the objects of my mission.

I was in entire ignorance of the best place to make my purchases, but having a letter to Messrs. Powers & Weightman, of Philadelphia, I was introduced by them to a large importing firm of that city, where I was fortunate enough to find a large stock of the very things I wanted. A representative of this firm had but recently returned from Europe, where he had visited all of the manufacturing cities of Germany and France and made a most beautiful and valuable collection of skeletons, models, charts, wax preparations and demonstration apparatus of every kind. Many of these articles are new and have never before been introduced into this country. I was fortunately enabled to avail myself of this collection. Finding this house to have everything I wanted, the entire purchase was made there, including chemicals, chemical and physiological apparatus, and a number of student's microscopes; in addition to which I ordered from Germany a magnificent microscope with all the latest improvements, to be used in the study of bacteriology.

I was doubtful when I left Texas, whether I would be able to find a gentleman competent to teach the above important branch, who could be induced to engage with us. In this matter also I was very successful. In asking the advice and assistance of the eminent pathologist, Dr. Shakespeare, I was told that Dr. Geo. Dock, assistant to Prof. Osler, of the University of Pennsylvania, was the only man in Philadelphia, who had received the proper training to teach bacteriology and cognate branches. I am happy to say that Dr. Dock has consented to come, and will sail for Texas early in October. Dr. Dock was in Leipzig the student of Reich. Hirchfield, in Berlin of Virchow, in Frankfort of Weigart. Since his return to America he has had charge of the pathological and bacteriological laboratories of the University of Pennsylvania. Dr. Dock's idea is to thoroughly ground every student in the practical use of the miscroscope, in histology and pathology, before attempting the more difficult department of bacteriology; hence we have secured a sufficient number of microscopes to enable every student to master these foundation studies.

I received upon every hand most encouraging words. Our efforts to place our school upon a high plane in the matter of a graded course, requirements for graduation, and, above all, the establishment of the above mentioned chair, met with the cordial endorsement of every physician whom I met in Philadelphia or New York.

Part of our goods have already arrived; a special importation made for us from Europe will reach us later, but will be here in ample time. Everything will be in readiness for the opening of the college in October. The Sealy hospital is not completed, but we will have all of the clinical material we need, in St. Mary's Infirmary, near the college.

The regents of the University, at a meeting lately held for the purpose, selected a plan for a new college building which will be complete in all of its appointments, and an ornament to the city. This, with the Sealy hospital and adjacent buildings, will make a most valuable plant for the State, when the University's finances are in such a condition as to render feasible the organization of a medical branch. In the mean time our efforts will be earnestly directed toward making the Texas Medical College and Hospital worthy the patronage of the physicians and medical students of the State.

Respectfully yours,

H. A. WEST, M. D.

---

[In addition to the above, we are tempted to publish the following extract from another letter from Dr. West, as we think it will be of interest to the readers of the JOURNAL.—ED.]

It is hardly necessary for me to say that I was delighted with Philadelphia. She truly deserves her title, the "City of Brotherly Love." I shall never forget the cordiality, hospitality and disinterested kindness with which I was treated on every hand. Especially will I remember Mr. Alex. H. Jones, of Powers & Weightman; Dr. Jas. Price, successor to Dr. Goodell, at the Preston Retreat, and Dr. Laurence Wolff, demonstrator of chemistry at Jefferson Medical College. I only hope that some fitting opportunity may occur when I may have the pleasure of reciprocating the numerous acts of kindness received at their hands.

Dr. Price is the most enthusiastic man I ever saw. Waking, he thinks and talks of nothing else, and sleeping, he dreams of *adominal section.* July, he tells me, was a very dull month,—he did only nineteen sections that month. I had the pleasure of witnessing two laparotomies which he dieected, while in Philadelphia. One was done as a last resort, in a case of septic peritonitis; the tubes were found to contain pus. The abdomen was well washed with boiled water; no iodoform or sublimate was used. The antisepsis consisted in absolute and perfect cleanliness. Dr. Price having been a most successful operator, and the number of abdominal sections done by him having been very large, it was quite interesting to me to observe the *technique* by which this success was attained, without the use of the foul smelling iodoform, or the much abused bichloride and carbolic acid solutions.

In the first place, he has two large, leather cases, each having a complete assortment of instruments and dressings which might be required in any case. By this means, in spite of frequent use, he is enabled to keep one set perfectly pure and clean. He uses none but the very best instruments, those which the experience of the largest operators have found to be of most perfect construction, and best fitted for their respective uses; the catch forceps, and several other instruments, are those perfected by Mr. Lawson Tait. One great advantage possessed by Dr. Price, and other Philadelphia surgeons, is the command they have of a number of trained nurses and assistants. Before an operation is to be done, one of them proceeds to the house, and thoroughly cleanses everything, from floor to ceiling; every recepticle for dirt is removed from the room; every article left is absolutely clean. While the woman is being etherized, the instruments to be used are immersed in boiling water, which is doubtless more effectual as a germ destroyer than the ordinary weak solutions of carbolic acid used to accomplish that result. The incision is made with a knife specially devised for that purpose, having a metal handle easy to keep clean. Dr. Price is a strong believer in a short incision, the advantages of which can readily be appreciated; for the educated finger, trained to do this work, but little space is required. By the short incision, many difficulties are avoided: protrusion, laceration, and chilling of omentum and intestine are prevented, weakness of the abdominal wall and subsequent hernia are not so apt to occur as when the incision is long. Dr. Price uses the drainage tube, in a majority of cases, and to it he attributes much of his success. It is hardly necessary to go into any further details upon this subject. After all, the difference between those who carry out antiseptic doctrines to the observance of the minutest detail, and those who claim to discard antisepticism *in toto*, is simply a difference in the agents used to accomplish the same purpose. Antisepsis is another name for *surgical cleanliness*, which is a very different thing from the term "cleanliness," as commonly used.

There are several things of interest I would like to mention, but as time is pressing, let this suffice.

Yours truly,

H. A. WEST.

